# Proceedings of the Pathological Society of Philadelphia

**Published:** 1861-03

**Authors:** 


PROCEEDINGS OF THE PATHOLOGICAL SOCIETY OF PHILADELPHIA.—We are in receipt of the first volume of the proceedings of this Society, an octavo of three hundred and seven pages, embracing the meetings of the body from the time of its organization, in October, 1857, up to the 8th of February, 1860. As the transactions originally appeared in the pages of the REVIEW, they are familiar to our readers, and it is, therefore, useless to say that they contain many valuable and interesting communications. Up to May 24th, 1860, the number of members was fifty, ten being corresponding, and forty resident members. This published volume bears sufficient evidence of the earnest purpose of the society, and shows that it is one of the best *working* medical organizations in Philadelphia.

